# CD40-TRAF Signaling Upregulates CX3CL1 and TNF-α in Human Aortic Endothelial Cells but Not in Retinal Endothelial Cells

**DOI:** 10.1371/journal.pone.0144133

**Published:** 2015-12-28

**Authors:** Jennifer A. Greene, Jose-Andres C. Portillo, Yalitza Lopez Corcino, Carlos S. Subauste

**Affiliations:** 1 Division of Infectious Diseases and HIV Medicine, Dept. of Medicine, Case Western Reserve University, Cleveland, Ohio, United States of America; 2 Department of Ophthalmology and Visual Sciences, Case Western Reserve University, Cleveland, Ohio, United States of America; 3 Department of Pathology, Case Western Reserve University, Cleveland, Ohio, United States of America; Cedars-Sinai Medical Center; UCLA School of Medicine, UNITED STATES

## Abstract

CD40, CX3CL1 and TNF-α promote atheroma and neointima formation. CD40 and TNF-α are also central to the development of diabetic retinopathy while CX3CL1 may play a role in the pathogenesis of this retinopathy. The purpose of this study was to examine whether CD40 ligation increases CX3CL1 and TNF-α protein expression in human endothelial cells from the aorta and retina. CD154 (CD40 ligand) upregulated membrane-bound and soluble CX3CL1 in human aortic endothelial cells. CD154 triggered TNF-α production by human aortic endothelial cells. TNF Receptor Associated Factors (TRAF) are key mediators of CD40 signaling. Compared to human aortic endothelial cells that express wt CD40, cells that express CD40 with a mutation that prevents TRAF2,3 recruitment, or CD40 with a mutation that prevents TRAF6 recruitment exhibited a profound inhibition of CD154-driven upregulation of membrane bound and soluble CX3CL1 as well as of TNF-α secretion. While both CD154 and TNF-α upregulated CX3CL1 in human aortic endothelial cells, these stimuli could act independently of each other. In contrast to human aortic endothelial cells, human retinal endothelial cells did not increase membrane bound or soluble CX3CL1 expression or secrete TNF-α in response to CD154 even though CD40 ligation upregulated ICAM-1 and CCL2 in these cells. Moreover, TNF-α did not upregulate CX3CL1 in retinal endothelial cells. In conclusion, CD40 ligation increases CX3CL1 protein levels and induces TNF-α production in endothelial cells. However, endothelial cells are heterogeneous in regards to these responses. Human aortic but not retinal endothelial cells upregulated CX3CL1 and TNF-α in response to CD40 ligation, as well as upregulated CX3CL1 in response to TNF-α. These dissimilarities may contribute to differences in regulation of inflammation in large vessels versus the retina.

## Introduction

Activated endothelial cells express various adhesion molecules and secrete chemokines that promote leukocyte recruitment and thus, foster inflammation [1]. Endothelial cell activation occurs in disorders with an inflammatory component including atherosclerosis and microvascular complications of diabetes [2,3]. Atherosclerosis is characterized by arterial lesions that contain infiltrating macrophages, T cells, dendritic cells and accumulation of cholesterol, collagen and proteoglycans [1]. Increasing evidence indicates that endothelial cells and low-grade chronic inflammation are also important in the pathogenesis of diabetic retinopathy [3].

CX3CL1 (fractalkine) is a chemokine that exists in a membrane-bound and a soluble form [4]. CX3CL1 is synthesized as a membrane-bound chemokine with a transmembrane domain and a chemokine domain positioned on top of a mucin-like stalk [4]. In this form, CX3CL1 promotes adhesion of monocytes, T cells and NK cells [5,6]. CX3CL1 is cleaved by A Disintegrin And Metalloprotease (ADAM)10 and ADAM17 [7,8]. Soluble CX3CL1 exhibits chemoattractant function for CX3CR1^+^ leukocytes [4]. The two forms of this molecule explain why CX3CL1 can have both adhesion molecule and chemokine functions.

Endothelial cells have low expression of CX3CL1 under resting conditions but upregulate this chemokine in response to inflammatory cytokines such as TNF-α and IFN-γ [9]. CX3CL1 is upregulated on endothelial cells in human atherosclerotic coronary arteries [10]. Moreover, this chemokine promotes the development of atherosclerosis. Atherosclerotic mice (ApoE^-/-^ or LDLR^-/-^) that lack CX3CR1 or CX3CL1 have decreased recruitment of macrophages to the vessel wall and reduced formation of atherosclerotic lesions [11–13]. CX3CL1 may have a pathogenic role in certain retinopathies. CX3CL1 appeared to promote retinal angiogenesis in mice with oxygen-induced retinopathy (OIR) [14], CX3CL1 levels are increased in the vitreous of patients with proliferative diabetic retinopathy [14] and CD11b^+^ cells in the retina of diabetic mice have increased CX3R1 expression [15].

TNF-α is well recognized for its pro-inflammatory effects on endothelial cells and likely plays a pivotal role in promoting endothelial cell dysfunction [16]. TNF-α is expressed in atheromas [17], and some studies suggest that this cytokine may promote atherosclerosis and neointima formation after arterial injury [18,19]. TNF-α has been reported to be upregulated in diabetic retinopathy [20,21] and promotes retinal leukostasis, blood-retinal barrier breakdown and capillary degeneration [21–23].

CD40 is another molecule that plays a key role in vascular inflammation. Although endothelial cells lack CD40 or express low levels of this molecule under basal conditions, CD40 is upregulated in endothelial cells during inflammation [24,25]. Engagement of endothelial cell CD40 with CD154 (CD40 ligand) upregulates various pro-inflammatory molecules [26–29]. CD154 increases steady state mRNA levels of CX3CL1 in human umbilical cord endothelial cells (HUVEC) [9]. CD40 plays a key role in the pathogenesis of atherosclerosis and diabetic retinopathy. CD40 is upregulated in endothelial cells in the atheroma, ApoE^-/-^ CD40^-/-^ mice exhibit diminished atherosclerotic lesions and CD40^-/-^ mice have decreased neointima formation after arterial injury [24,25,30,31]. Similarly, CD40 is upregulated in retinal endothelial cells in mice with diabetic retinopathy and diabetic CD40^-/-^ mice do not develop retinopathy [32].

Herein we studied whether CD40 ligation in endothelial cells upregulates membrane and soluble CX3CL1 protein expression and induces TNF-α production. There is functional heterogeneity among endothelial cells [33,34]. We focused on human aortic endothelial cells (HAEC) and human retinal endothelial cells (HREC) to determine whether these responses may be affected by the tissue of origin of endothelial cells. We also examined the role of TNF Receptor Associated Factors (TRAF) in the regulation of CX3CL1 and TNF-α expression. TRAFs are important mediators of CD40 signaling that are recruited to TRAF2,3 and TRAF6 binding sites in the intracytoplasmic tail of CD40 upon CD40 –CD154 interaction [35–37]. Our studies revealed that CD40 ligation in HAEC induces marked upregulation of CX3CL1 and secretion of TNF-α. These responses required both the TRAF2,3 and TRAF6 binding sites of CD40. In marked contrast, HREC were unable to upregulate CX3CL1 and secrete TNF-α in response to CD40 stimulation and did not increase CX3CL1 expression after incubation with TNF-α. These findings may contribute to the more restricted nature of inflammation in the retina compared to large blood vessels.

## Materials and Methods

### Cells and *in vitro* stimulation

Primary HAEC and HUVEC obtained from Lonza (Allendale, NJ) and A.T.C.C. (Manassas, VA) were cultured following supplier’s recommendations. Human eyes from 3 non-diabetic donors were obtained from the Cleveland Eye Bank. Primary HREC were obtained as described [38]. Their identity was confirmed by incorporation of acetylated low-density lipoprotein and CD31 staining (> 95%). HREC were cultured in gelatin-coated tissue culture flasks containing DMEM, 10% FBS (HyClone, Logan, UT), endothelial cell growth supplement from bovine pituitary (15μg/ml; Sigma Chemical, St Louis, MO) and insulin/transferrin/selenium (Sigma Chemical). Growth factors were removed from culture media 24 h prior to stimulation with CD154. All endothelial cells were > 95% viable (trypan blue exclusion) throughout experiments. Endothelial cells were stimulated with cell-free supernatants containing multimeric CD154 obtained from Dr. Richard Kornbluth, Multimeric Biotherapeutics Inc., La Jolla, CA) for 24 h at 37°C as described [39]. Omission of CD154 or incubation with a non-functional CD154 mutant (T147N; obtained from Dr. Richard Kornbluth) were used as controls. Cells were also incubated with recombinant soluble CD154 (1 μg/ml) plus enhancer (2 μg/ml) (Enzo Life Sciences; Farmingdale, NY) or with human TNF-α (20 ng/ml unless stated otherwise; Peprotech, Rocky Hill, NJ). In certain experiments cells were incubated with a neutralizing anti-human CD154 mAb (Ancell Corporation, Bayport, MN), neutralizing anti-human TNF-α mAb (R & D Systems, Minneapolis, MN), control mAb (BD Biosciences, San Jose, CA; all at 10 μg/ml) or GM 6001 (50 μM; Tocris Bioscience, Bristol, UK). Reagents were devoid of endotoxin as assessed by limulus amebocyte lysate assay (< 0.01 EU/ml).

### Retroviral vectors

MIEG3-based retroviral vectors that encode enhanced GFP (EGFP) and either cDNA for wt human CD40, CD40 ΔTRAF2,3, CD40 ΔTRAF6, or CD40 ΔTRAF2,3,6 were previously described [40]. These vectors were generated using cDNA for wild-type human CD40 (hCD40), hCD40Δ22 (a mutant that ablates binding to TRAF2 and TRAF3; CD40 ΔTRAF2,3), hCD40EEAA (a mutant that prevents binding to TRAF6; CD40 ΔTRAF6), and hCD40Δ55 (a mutant that ablates binding to TRAF2, TRAF3 and TRAF6; CD40 ΔTRAF2,3,6) [41,42]. Ecotropic retroviral supernatants were generated as described [43]. Endothelial cells were incubated overnight with retrovirus in the presence of polybrene (8 μg/ml, Sigma Chemical).

### Flow cytometry

Surface staining was performed as described previously [44]. Cells were stained with PE-labeled CX3CL1 (R&D Systems, Minneapolis, MN), PE-labeled CD40 (BD Biosciences), PE-labeled ICAM-1 (BD Biosciences) or PE-labeled isotype control mAbs. Cells were fixed in 1% PFA and analyzed on an LSR II flow cytometer (BD Biosciences, Franklin Lakes, NJ). Mean fluorescence intensity of isotype controls was subtracted from each sample to determine corrected mean fluorescence intensity (cMFI).

### ELISA

Concentrations of CX3CL1, TNF-α and CCL2 in supernatants were determined by ELISA (R&D Systems and eBioscience, San Diego, CA) according to manufacturer’s instructions.

### Statistical analysis

Statistical analysis was performed with GraphPad Prism 4 (La Jolla, CA). Each experiment was repeated at least two additional times, and statistics were run on the total of all the data. Statistical significance was determined by Student’s *t* test and Analysis of Variance.

## Results

### CD40 ligation upregulates membrane CX3CL1 on human aortic endothelial cells and human umbilical vein endothelial cells

**Fig 1:**
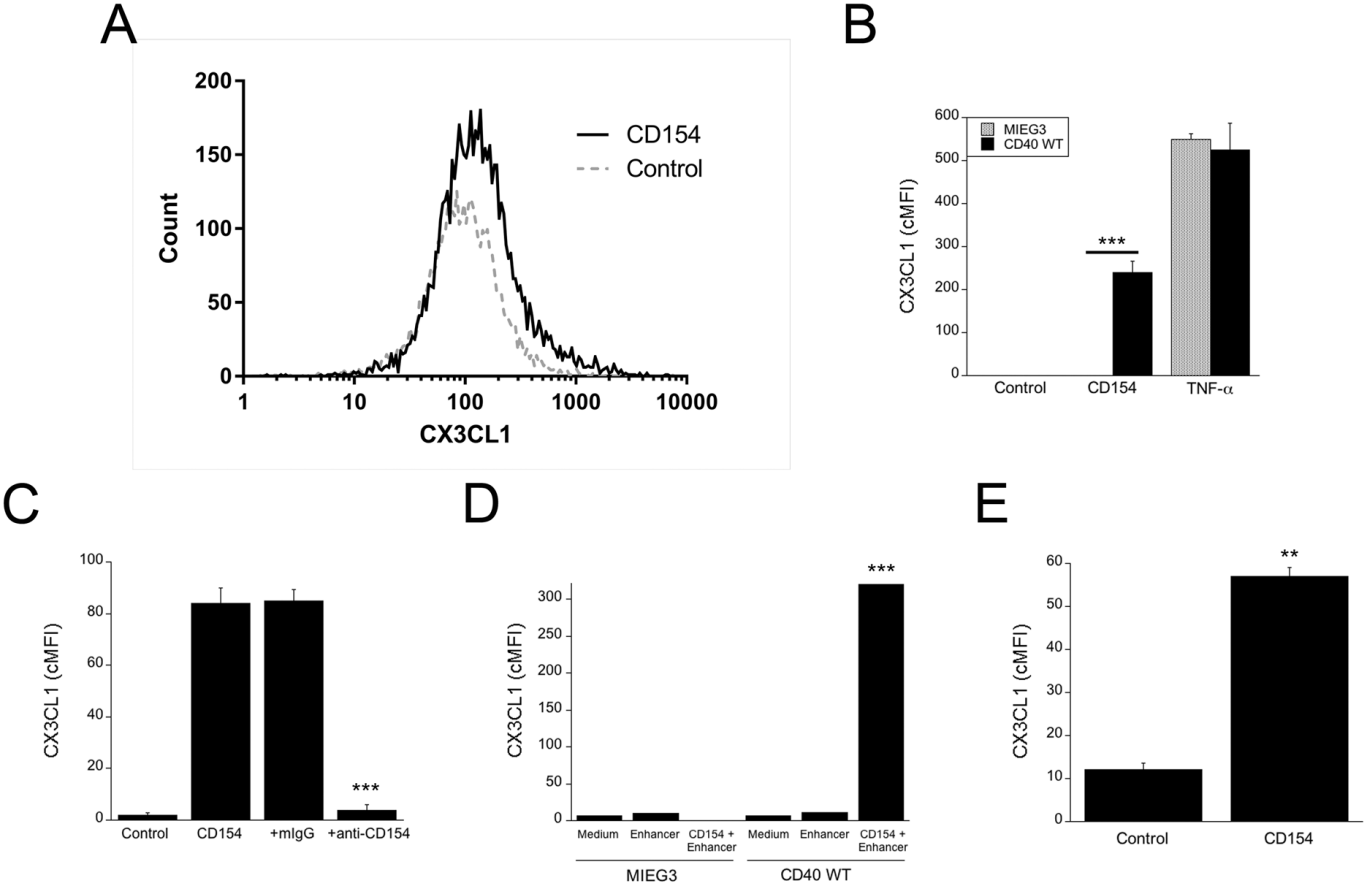
CD40 ligation upregulates membrane CX3CL1 in HAEC and HUVEC. (A–D) HAEC were transduced with MIEG3-based retroviral vector that encode EGFP or EGFP plus wt CD40. CX3CL1 expression was analyzed on transduced (EGFP^+^) cells. (A) Histograms show CX3CL1 expression on HAEC transduced with wt CD40-encoding retroviral vector at 24 h post-incubation with or without multimeric CD154. (B) Expression of CX3CL1 (cMFI) on gated EGFP^+^ cells after incubation with or without multimeric CD154 or TNF-α. (C) Transduced HAEC that express wt CD40 were incubated with multimeric CD154 with either anti-CD154 or isotype control mAb. (D) Transduced HAEC that express wt CD40 were incubated with recombinant CD154 with or without enhancer used to crosslink CD154. (E) HUVEC were incubated with or without multimeric CD154. Results are shown as mean ± SEM and are representative of 3–5 experiments. ** p < 0.01; *** p < 0.001.

We examined whether CD40 ligation upregulates membrane bound CX3CL1 on HAEC. While endothelial cells express CD40 in the setting of inflammation, HAEC lack detectable CD40 under resting conditions. We induced expression of CD40 in these cells using retroviral vectors, an approach that has been shown to be not only adequate to examine pro-inflammatory responses induced by CD40 in primary cells [43,45] but also allows to determine the relative role of TRAF signaling cascades in mediating the effects triggered by CD40 ligation [42,43,45–48]. HAEC were transduced with MIEG3-based retroviral vector encoding wild type CD40. This is a bicistronic vector that also encodes EGFP allowing the identification of transduced cells. The levels of CX3CL1 were examined in the transduced populations. Incubation with CD154 upregulated membrane CX3CL1 in HAEC transduced with the wt CD40 vector (Fig 1A and 1B). CX3CL1 upregulation was unimodal and thus, CX3CL1 levels are expressed as cMFI. Control HAEC transduced with the empty vector did not upregulate membrane CX3CL1 in response to incubation with CD154 (Fig 1B). In contrast, both HAEC transduced with the empty or the wt CD40 vector upregulated CX3CL1 in response to TNF-α (Fig 1B). Addition of a neutralizing anti-CD154 mAb confirmed that the effect of CD154 was specific (Fig 1C). Similar results were observed when HAEC that express wt CD40 were incubated with commercial recombinant CD154 (Fig 1D).

Next, we tested the effect of CD154 on endothelial cells that express basal levels of CD40. In contrast to HAEC, HUVEC express detectable levels of CD40 under basal conditions (CD40 cMFI 100). CD154 upregulated membrane CX3CL1 in HUVEC (Fig 1E). These results indicate that upregulation of CX3CL1 is not restricted to endothelial cells with retroviral-driven CD40 expression. Taken together, CD40 ligation upregulates membrane CX3CL1 in HAEC and HUVEC.

### CD40 ligation stimulates CX3CL1 secretion by human aortic endothelial cells and human umbilical vein endothelial cells

**Fig 2:**
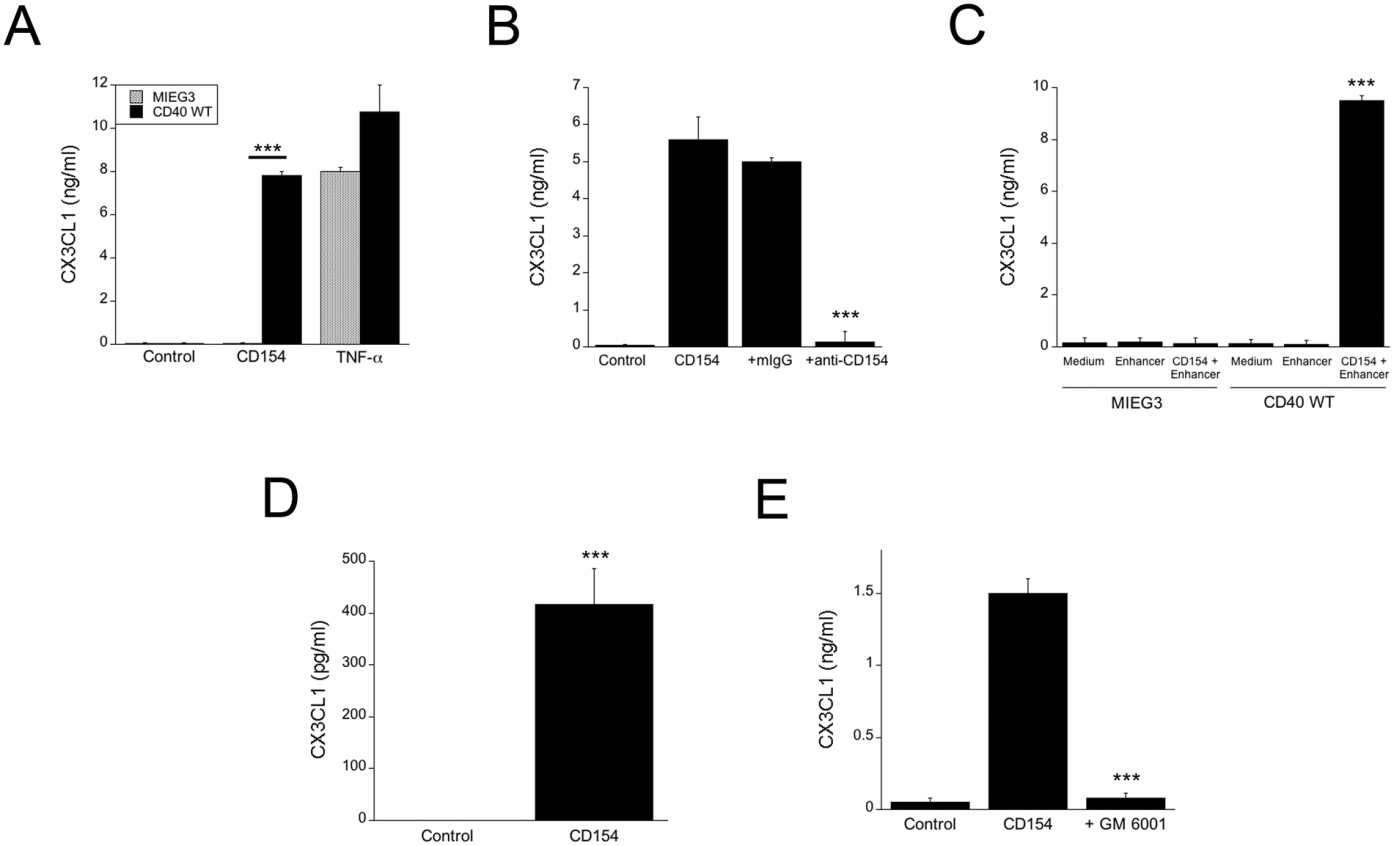
CD40 ligation stimulates CX3CL1 secretion by HAEC and HUVEC. (A) HAEC transduced with control or wt CD40 retroviral vector were incubated with or without multimeric CD154 or TNF-α for 24 h. CX3CL1 concentrations in supernatants were determined by ELISA. (B) Transduced HAEC that express wt CD40 were incubated with multimeric CD154 with either anti-CD154 or isotype control mAb. (C) HAEC transduced with control or wt CD40 retroviral vector were incubated with recombinant CD154 with or without enhancer. (D) HUVEC were incubated with or without multimeric CD154. (E) Transduced HAEC that express wt CD40 were incubated with multimeric CD154 with or without the MMP inhibitor GM 6001. CX3CL1 concentrations in supernatants were determined by ELISA. Results are shown as mean ± SEM and are representative of 3–5 experiments. *** p < 0.001.

Upregulation of membrane bound CX3CL1 may not be necessarily accompanied by increased release of soluble CX3CL1. For example, in contrast to TNF-α, IFN-γ upregulates membrane bound CX3CL1 in endothelial cells but does not efficiently promote release of the soluble form of this chemokine [9]. Incubation with CD154 upregulated secretion of soluble CX3CL1 by HAEC transduced with the wt CD40 vector but not by control HAEC transduced with the empty vector (p < 0.001) (Fig 2A). In contrast, both HAEC transduced with the empty or the wt CD40 vector secreted CX3CL1 in response to TNF-α (Fig 2A). Similar to membrane CX3CL1, the effect of CD154 was specific since it was neutralized by anti-CD154 mAb (p < 0.001) (Fig 2B), and was confirmed by incubation with commercial recombinant CD154 (p < 0.001) (Fig 2C). CD154 also triggered secretion of CX3CL1 by HUVEC (Fig 2D).

Soluble CX3CL1 is generated by cleavage of the membrane bound chemokine by the matrix metalloproteinases (MMP) ADAM10 and ADAM17. We used GM 6001, a broad spectrum MMP inhibitor, to test if CD40-mediated soluble CX3CL1 expression was dependent on MMP function. Addition of GM 6001 significantly reduced the amount of soluble CX3CL1 detected in the cell supernatant (Fig 2E). Taken together, CD40 ligation not only upregulated membrane CX3CL1 but also increased secretion of this chemokine in HAEC and HUVEC.

### Both the CD40-TRAF2,3 and the CD40-TRAF6 binding sites are required for CD154-induced upregulation of CX3CL1 in human aortic endothelial cells

**Fig 3:**
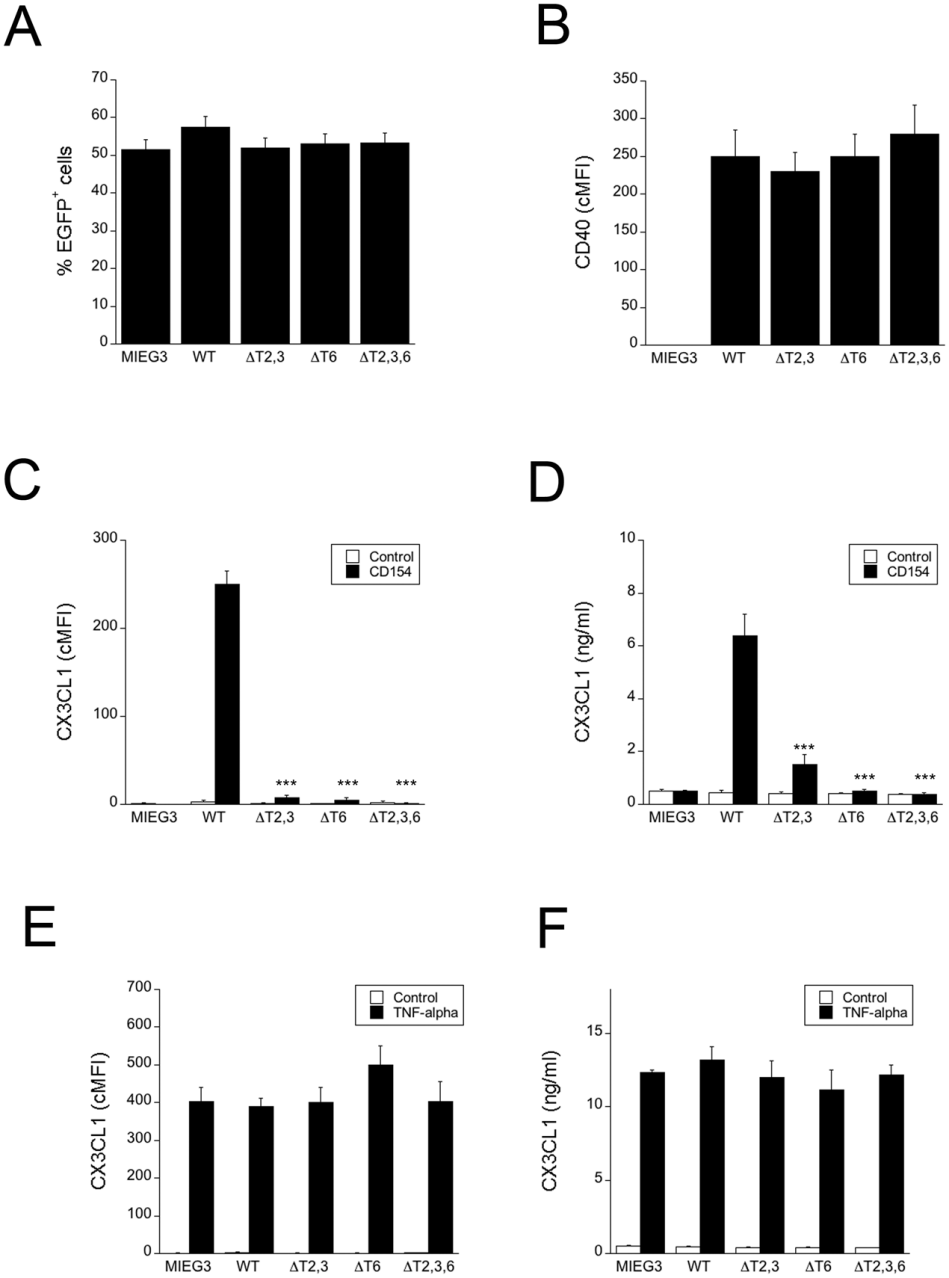
Both the CD40-TRAF2,3 and CD40-TRAF6 binding sites are required for optimal CX3CL1 upregulation in HAEC. HAEC were transduced with MIEG3-based retroviral vector that encode EGFP and either wt CD40, CD40 ΔT2,3, CD40 ΔT6, CD40 ΔT2,3,6. (A) Percentages of HAEC that became EGFP^+^ after incubation with retroviral vectors. (B) Expression of CD40 on gated EGFP^+^ cells shown as cMFI. (C, D) HAEC transduced with the retroviral vectors were incubated with or without multimeric CD154 for 24 h. Expression of CX3CL1 (cMFI) on gated EGFP^+^ cells was assessed by flow cytometry (C) and CX3CL1 concentrations in supernatants were determined by ELISA (D). (E, F) HAEC transduced with the retroviral vectors were incubated with or without TNF-α for 24 h. Expression of CX3CL1 (cMFI) on gated EGFP^+^ cells was assessed by flow cytometry (E) and CX3CL1 concentrations in supernatants were determined by ELISA (F). Results are shown as mean ± SEM and are representative of 3 experiments. *** p < 0.001.

In order to examine the role of CD40 –TRAF signaling in CX3CL1 upregulation, HAEC were transduced with retroviral vectors that encode either wt CD40 or CD40 with deletions or point mutations at TRAF binding sites proven to ablate binding to TRAF2,3 (ΔT2,3), TRAF6 (ΔT6) or TRAF2,3,6 (ΔT2,3,6) [42,46]. HAEC transduced with wild type or mutant CD40 had similar levels of transduction efficiency (% EGFP^+^) and CD40 expression (Fig 3A and 3B). CD154 upregulated both membrane bound and soluble CX3CL1 in HAEC that express wt CD40 (Fig 3C and 3D). When either the TRAF2,3 or the TRAF6 site was mutated, both membrane bound and soluble CX3CL1 expression were markedly decreased. In contrast, expression of membrane bound and soluble CX3CL1 induced by TNF-α were similar in all groups of cells (Fig 3E and 3F). Thus, mutation in either the TRAF2,3 or the TRAF6 binding sites was sufficient to markedly impair CX3CL1 upregulation.

### CD154 induces TNF-α secretion by human aortic endothelial cells in a CD40-TRAF2,3 and CD40-TRAF6-dependent manner

**Fig 4:**
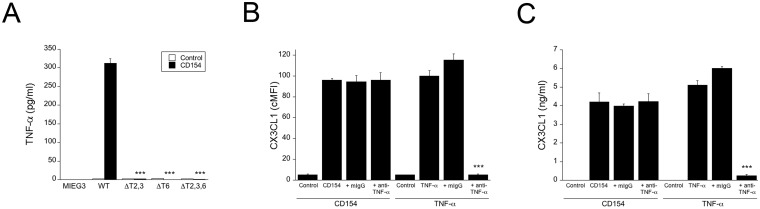
CD40 ligation upregulates TNF-α in HAEC in a CD40-TRAF2,3 and CD40-TRAF6-dependent manner. (A) HAEC were transduced with retroviral vectors as indicated followed by incubation with or without multimeric CD154 for 24 h. TNF-α concentrations in supernatants were determined by ELISA. (B, C) HAEC that express wt CD40 were incubated with multimeric CD154 or TNF-α in the presence of a neutralizing anti-TNF-α or control mAb. Expression of membrane bound (B) and soluble CX3CL1 (C) were assessed by flow cytometry and ELISA respectively. Results are shown as mean ± SEM and are representative of 3 experiments. *** p < 0.001.

HAEC were transduced with retroviral vectors encoding wild type or mutant CD40. CD154 triggered TNF-α production in HAEC that expressed wt CD40 (Fig 4A). Similar results were obtained with commercial recombinant CD154 (not shown). Mutations in either the TRAF2,3 or TRAF6 binding sites ablated secretion of this cytokine (Fig 4A). Next, we examined whether TNF-α mediates CX3CL1 upregulation in HAEC. HAEC that express wt CD40 were incubated with CD154 in the presence or absence of a neutralizing anti-TNF-α mAb. Fig 4B and 4C shows that anti-TNF-α mAb did not affect upregulation of CX3CL1 induced by CD154. In contrast, anti-TNF-α inhibited TNF-α -mediated CX3CL1 upregulation (Fig 4B and 4C). Thus, CD40 ligation induces TNF-α secretion by HAEC. However, autocrine TNF-α does not mediate CX3CL1 upregulation.

### Human retinal endothelial cells do not secrete CX3CL1 or TNF-α in response to CD154 and do not upregulate CX3CL1 in response to TNF-α

**Fig 5:**
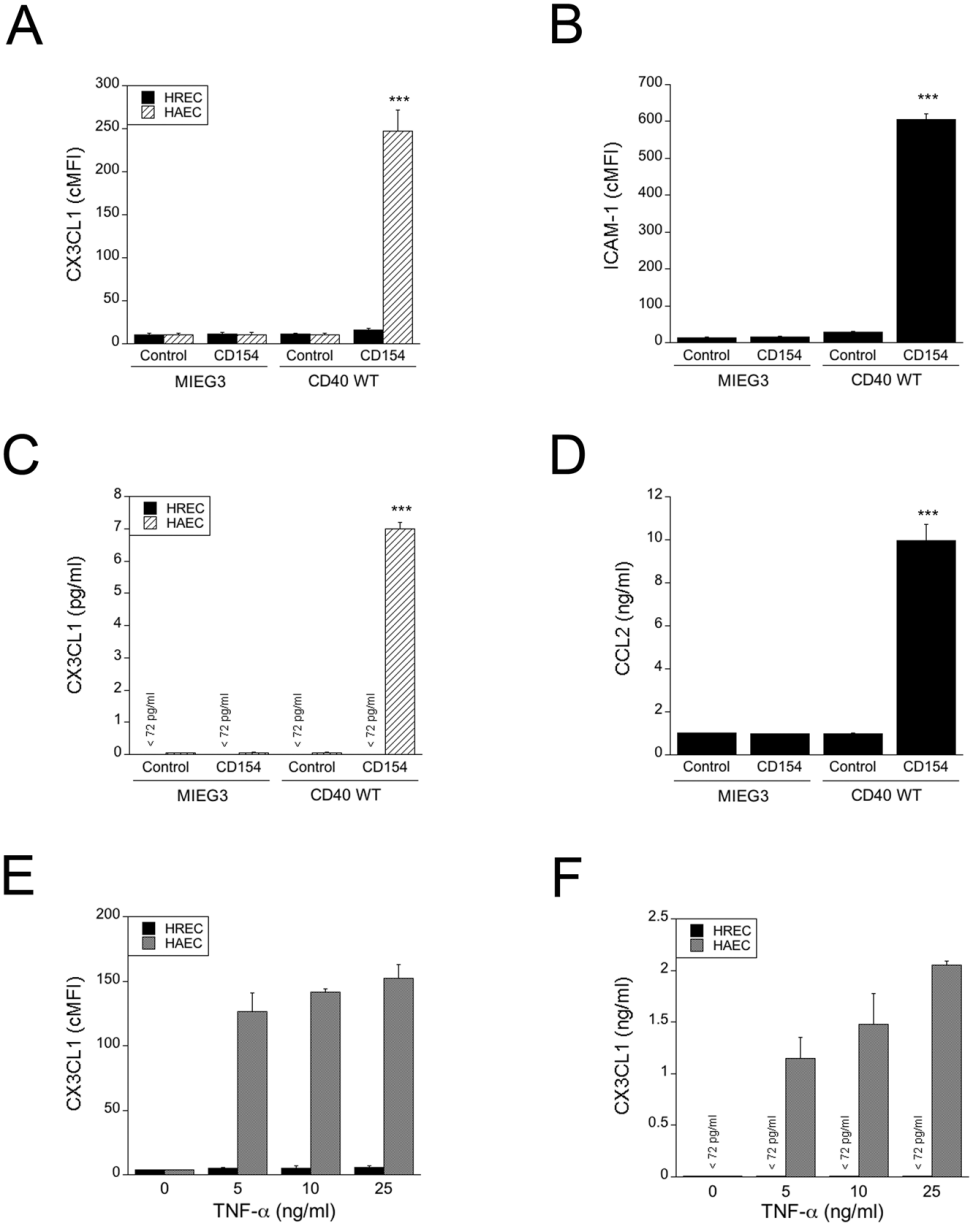
HREC do not upregulate CX3CL1 in response to CD40 ligation or TNF-α and do not secrete TNF-α after CD40 ligation. (A–D) HREC and HAEC transduced with empty retroviral vector or retroviral vector that encodes wt CD40 were incubated with or without multimeric CD154 for 24 h. (A) Expression of membrane-bound CX3CL1 was assessed by flow cytometry. (B) Expression of ICAM-1 on HREC was assessed by flow cytometry. (C) CX3CL1 concentrations in supernatants were determined by ELISA. (D) CCL2 concentrations in supernatants from HREC were determined by ELISA. (E–F) Untransduced HAEC and HREC were incubated with TNF-α as indicated for 24 h. Expression of membrane-bound (E) and soluble CX3CL1 (F) were assessed by flow cytometry and ELISA respectively. Results are shown as mean ± SEM and are representative of 4 experiments. *** p < 0.001.

HREC were transduced with the retroviral vector that encodes wt CD40 or the empty vector. The percentages of transduced cells were on average 55% ± 8.6% and were similar in both groups (p > 0.05). cMFI for CD40 in HREC transduced with the wt CD40-encoding vector was on average 184.3 ± 13.2. In contrast to HAEC and HUVEC, CD154 did not cause detectable upregulation of membrane CX3CL1 or secretion of soluble CX3CL1 in HREC that express wt CD40 (Fig 5A and 5C). CD40 is functional in HREC since, as previously reported [45], CD154 upregulated ICAM-1 and induced CCL2 secretion in HREC expressing wt CD40 (Fig 5B and 5D). Moreover, HREC that express wt CD40 do not secrete detectable amounts of TNF-α in response to CD154 (< 7.8 pg/ml; data not shown). Next, we examined whether TNF-α could upregulate CX3CL1 in HREC. HREC and HAEC were incubated with increasing concentrations of TNF-α. While TNF-α upregulated CX3CL1 in HAEC, this cytokine did not cause detectable upregulation of CX3CL1 in HREC (Fig 5E and 5F). Similar results were obtained regardless of the donor for REC. Taken together, these findings indicate that in contrast to HAEC, HREC do not upregulate CX3CL1 and TNF-α after CD40 ligation and HREC failed to upregulate CX3CL1 in response to TNF-α.

## Discussion

We report that CD40 ligation in HAEC increases expression of the membrane-bound and the soluble forms of CX3CL1, and triggered TNF-α secretion. The upregulation CX3CL1 and TNF-αwere dependent on TRAF signaling. Interestingly, both the CD40—TRAF2,3 and the CD40—TRAF6 pathways needed to be functional in order for marked upregulation of CX3CL1 and TNF-α to occur. CD40 stimulation in HUVEC also upregulated membrane and soluble CX3CL1. In marked contrast, HREC did not upregulate CX3CL1 and did not secrete TNF-α in response to CD40 ligation. HREC also failed to upregulate CX3CL1 when incubated with TNF-α. These findings revealed that CD40 –TRAF signaling induces CX3CL1 protein expression and TNF-α secretion in endothelial cells and the organ of origin of the endothelial cells appears to affect these responses. These apparent regional changes in CD40-driven induction of CX3CL1 and TNF-α in endothelial cells may contribute to differences in the regulation of leukocyte recruitment in large vessels and the retina.

CX3CL1 upregulation in endothelial cells from large vessels has been demonstrated to cause increased adhesion of leukocytes [5]. Although not directly tested in our studies, it is likely that CD40-induced CX3CL1 upregulation could promote adhesion of CX3CR1^+^ monocytes. CD40 and CX3CL1 have a well-established role in the pathogenesis of vascular inflammatory disorders such as atherosclerosis and neointima formation after arterial injury [11–13,24,25,30,31]. Monocytes express CX3CR1 and recruitment of monocytes to the vascular wall is an important event in the pathogenesis of vascular inflammation [1]. While the studies presented herein were done *in vitro*, our findings raise the possibility that CD40-mediated CX3CL1 upregulation in endothelial cells may be relevant *in vivo* by promoting monocyte recruitment during atherosclerosis or arterial injury-driven neointima formation. Moreover, our studies suggest that CD40-driven secretion of TNF-α by HAEC may contribute to endothelial dysfunction during vascular inflammation.

Expression of wt CD40 or CD40 with mutations that prevent recruitment of TRAF2,3 and/or TRAF6 to CD40 allows to study the role of selective TRAF signaling in cellular responses triggered by CD40 ligation. After confirming that no significant differences in CD40 expression were detected among cells that expressed wt CD40 or CD40 mutants, this approach enabled us to establish the critical role of CD40-TRAF signaling for the upregulation of CX3CL1 and TNF-α by HAEC. Importantly, mutation in TRAF2,3 or TRAF6 binding sites was sufficient to cause a profound inhibition in CX3CL1 upregulation and TNF-α production. We previously reported that the TRAF2,3 and TRAF6 binding sites cooperate to promote CD40-driven upregulation of ICAM-1, VCAM-1, CCL2, MMP-9 and tissue factor in HAEC [43]. It appears that in the case of CX3CL1 and TNF-α the presence of functional TRAF2,3 and TRAF6 binding sites is a requirement since mutation in either site was sufficient to cause 82% to 100% inhibition in CX3CL1 and TNF-α upregulation.

Endothelial cells are heterogeneous in regards to their function, phenotype and responses to inflammatory stimuli [33,34]. It appears that some of this heterogeneity may be explained by the donor source of the endothelial cells. For example, while all HUVEC preparations isolated from 30 different donors secreted IL-8 in response to endotoxin, the level of response appeared to vary among donors [49]. It would appear unlikely that donor genetic factors explained the contrasting pro-inflammatory responses noted between REC and endothelial cells from large vessels since similar results were obtained regardless of the donor. The profound differences noted suggest that there are more fundamental differences in the capacity of these endothelial cells to upregulate CX3CL1 and TNF-α Of relevance, *in vivo* administration of TNF-α increased CX3CL1 expression in arterial and capillary endothelial cells in mice, whereas little or no induction of CX3CL1 was observed in venous and lymphatic endothelial cells [50]. Functional heterogeneity for CX3CL1 upregulation may also apply to endothelial cells in different compartments of the eye. Studies that used enzyme-linked immunoculture assay in monolayers of human iris endothelial cells suggested that CD154 may upregulate CX3CL1 in these cells [51]. Functional heterogeneity among endothelial cells is explained, at least in part, by differences in transcriptional regulation [33]. The inability of two stimuli, CD40 and TNF-α, to trigger detectable upregulation of CX3CL1 in REC raises the possibility that there may be differences in basic mechanisms of CX3CL1 transcriptional regulation between REC and endothelial cells from large vessels.

Our findings in HAEC and HREC are in accordance with the different regulation of inflammation in large arteries and the retina. Expression of membrane-bound CX3CL1 in endothelial cells directly promotes leukocyte attachment in low shear conditions [5]. Endothelial CX3CL1 also promotes leukocyte adhesion at higher shear forces, an effect that is mediated by CX3CL1-driven platelet activation, followed by release of P-selectin and leukocyte attachment promoted by P-selectin [52]. The upregulation of CX3CL1 in HAEC is in agreement with the notion that CX3CL1-mediated attachment and recruitment of leukocytes is operative in vascular inflammation associated with atherosclerosis and arterial injury. In contrast, the lack of CX3CL1 upregulation in HREC in response to CD40 ligation and TNF-α may be in accordance with the fact that the retina is an organ where inflammation is more tightly controlled in order to minimize tissue injury. The evidence that CD11b^+^ cells in the retina of diabetic mice have increased CX3R1 expression [15] and the upregulation of CX3CL1 in the vitreous of mice with OIR [14] suggest that this chemokine plays a pathogenic role in certain retinopathies. Our studies suggest the importance of non-endothelial sources of CX3CL1 in the retina. Indeed, CX3CL1 expression has been detected in retinal neurons and likely Muller cells [51,53]. In this scenario, CX3CL1 may have a more restricted function in leukocyte recruitment since it would not directly mediate leukocyte attachment to retinal endothelial cells. Of relevance, studies in ischemic brain revealed that endothelial cells expressed CCL2 while expression of CX3CL1 occurred in astrocytes [54]. Similarly, the lack of detectable TNF-α secretion by CD40-stimulated HREC may also assist in restricting inflammatory responses in the retina. Future studies that examine whether CD40 ligation in Muller cells induces CX3CL1 and/or TNF-α upregulation may provide further insight into the regulation of expression of these molecules in the retina.

CD40 can synergize with TNF-α or IL-1β to induce certain cellular responses. For example, CD40 synergizes with TNF-α to upregulate NOS2 in mouse macrophages, an effect that my be explained by convergence of TRAF6 signaling downstream of CD40 with CEBP-γ signaling downstream of TNF receptor [55]. CD40 triggers autocrine production of TNF-α that in turn synergizes with signals downstream of CD40 to stimulate autophagy [40,56]. In addition, CD40 synergizes with IL-1β to enable retinal endothelial cells to secrete KC/CXCL1 [39]. Our studies indicate that while CD40 induces autocrine TNF-α in HAEC, this cytokine does not drive CX3CL1 upregulation after CD40 stimulation of HAEC. These results may be explained by the observation that CD40 stimulation of HAEC releases concentrations of TNF-α that are lower than those required to upregulate CX3CL1.

Studies in animals revealed that administration of blocking anti-CD154 mAb was effective against various inflammatory disorders [57]. Unfortunately, clinical trials of anti-CD154 mAb administration in humans encountered thromboembolic events that appeared not to be cause by inhibition of CD40 –CD154 signaling per se [58]. Inhibition of signaling downstream of CD40 is an alternative approach to inhibit the CD40-CD154 pathway. However, global inhibition of CD40 signaling is predicted to cause immunosuppression. In this regard, IL-12 secretion by dendritic cells, dendritic cell maturation, TNF-α, IL-1β, IL-6 and NOS2 upregulation in macrophages, and autophagy-mediated anti-microbial activity, IL-6 production by B cells, affinity maturation and the generation of long lived plasma cells are mediated exclusively or predominantly by CD40 –TRAF6 signaling [43,48,55,56,59–62]. On the other hand, the CD40-TRAF2,3 signaling promotes isotype switch [47]. The observation that blockade of CD40-TRAF2,3 signaling is sufficient to markedly inhibit CD40-driven upregulation of CX3CL1 and TNF-α in HAEC supports the increasing evidence that this pathway plays a central role in the stimulation of pro-inflammatory responses in non-hematopoietic cells [43,45]. These findings together with the demonstration that non-hematopoietic cells are crucial to the development of inflammatory disorders [25,39] suggest that inhibition of CD40-TRAF2,3 signaling may prove to be an effective approach to control CD40-driven inflammation while at the same time minimizing the risk of immunosuppression.

The heterogeneity of endothelial cells from different vascular beds impacts the response of these cells to stimuli that trigger inflammation [50,63]. In turn, the patterns of expression of pro-inflammatory molecules likely modulate leukocyte recruitment. The differences noted in this study in the upregulation of CX3CL1 and TNF-α in endothelial cells may explain in part contrasts in susceptibility to inflammation between large blood vessels and the retina.

## Abbreviations

ADAM: A Disintegrin And Metalloprotease
cMFI: corrected mean fluorescence intensity
EGFP: enhanced green fluorescence protein
HAEC: human aortic endothelial cells
HUVEC: human umbilical vein endothelial cells
HREC: human retinal endothelial cells
MMP: metalloproteinase
OIR: oxygen-induced retinopathy
TRAF: TNF receptor associated factor
